# Dielectrophoretic capture of *Escherichia coli* and boar sperms using ULSI-fabricated three-dimensional protruding TiN nano-electrode arrays

**DOI:** 10.3389/fbioe.2024.1470606

**Published:** 2024-10-01

**Authors:** Hua-Jung Lu, I-Hsuan Liao, Chun-Lung Lien, Jeng-Huei Shiau, Ching-Fen Shen, Kuan-Ru Chou, Chao-Min Cheng

**Affiliations:** ^1^ Institute of Biomedical Engineering, National Tsing Hua University, Hsinchu, Taiwan; ^2^ NEAT Biotech Inc., Hsinchu, Taiwan; ^3^ Department of Pediatrics, National Cheng Kung University Hospital, College of Medicine, National Cheng Kung University, Tainan, Taiwan

**Keywords:** dielectrophoresis, nano-electrode arrays, CMOS, titanium nitride, *Escherichia coli* (*E. coli*), boar sperm

## Abstract

**Object:**

In recent years, dielectrophoresis has become widely recognized as a highly suitable method for creating good tools for particle separation, with significant successes achieved in a variety of areas.

**Method:**

Expanding upon this, we adopted a semiconductor CMOS process, instead of a MEMS process, which allowed for the following: 1) wire insulation to mitigate Joule heat and prevent thermal fluctuation interference with the dielectrophoretic force; 2) isolation of harmful materials from biological samples, making the chip biocompatible; and, 3) the ability to employ nano-electrodes capable of generating a stronger electric field than conventional electrodes, thus allowing chip capture at lower voltages. Additionally, our chip is scalable, enabling multiplied throughput based on sample processing requirements.

**Results and Dissusion:**

These features make our chip more widely applicable and suitable for capturing bacteria and sperm. In this study, we focused on optimizing the parameters of dielectrophoresis and employed 3-D protruding TiN nano-electrode arrays to facilitate the capture of *Escherichia coli* and boar sperms. The experimental data demonstrates that the capture efficiency of this chip for *E. coli* was approximately 79.25% ± 2.66%, and the highest capture efficiency for sperms was approximately 39.2% ± 3.9%.

## Introduction

Dielectrophoresis is a phenomenon in which a polarizable particle suspended in a non-uniform electric field experiences a force that causes it to move toward a specific direction. The force is caused by the induced asymmetric polarization of the particles. The direction of the dielectrophoretic force depends on the polarizability of the particle relative to that of the surrounding medium. According to the dielectrophoresis Equation 1, the magnitude of the dielectrophoretic force is related to Clausius-Mossotti (CM) factor, which is influenced by the dielectric constants of both particle and medium, as shown in the Equation 2. Here, $\varepsilon_{p}^{*}$ and $\varepsilon_{m}^{*}$ represent the dielectric constants of the particle and the medium respectively, *r* means a radius of the spherical particle, $Re\left[CM\left(f\right)\right]$ means the real part Clausius-Mossotti factor, f is the frequency of electric field, ${\left(\nabla E\right)}_{RMS}^{2}$ is the root-mean-square value of the gradient of the applied electric field, where ∇E represents the gradient of the electric field. In the equation, the dielectric constant represents the particle’s polarizability, with a higher dielectric constant indicating greater polarizability (Ronald, 2010; Jones, 1995; Zhang et al., 2019). If the particle is more polarizable than the medium, a positive value for CM indicates that the particle will be attracted to highest field region which means positive dielectrophoresis (Ronald, 2010). Conversely, if the particle is less polarizable than the medium, it will be repelled from the highest field region (negative dielectrophoresis). Dielectrophoresis has been used for a variety of applications involved with the separation and manipulation of biological molecules, cells, DNA, and bacteria. It is a powerful tool for label-free separation and can be used to isolate specific biomolecules from complex mixtures (Ronald, 2010).

$$F_{DEP}=2\pi \varepsilon_{m}\varepsilon_{p}r_{ext}^{3}Re\left[CM\left(f\right)\right]{\left(\nabla E\right)}_{RMS}^{2}$$ (1)

$$CM\left(f\right)=\left[\frac{\varepsilon_{p}^{*}-\varepsilon_{m}^{*}}{\varepsilon_{p}^{*}+{2\varepsilon }_{m}^{*}}\right]$$ (2)

In recent years, the application of dielectrophoresis has become increasingly widespread, with technological advancements progressing rapidly. Many satisfactory results have been achieved in various fields, including cell therapy, biosensing, medical diagnosis, drug development, and particle separation (Jubery et al., 2014; Bashir and Li, 2002). To optimize the application of dielectrophoresis, many experts are exploring different materials and device designs to confirm the biocompatibility and feasibility of dielectrophoresis. In the past, dielectrophoresis-based chips are mostly fabricated through using MEMS technology. Their structures are fabricated using standard BEOL metal patterning processes, which are typically limited to the micron-level feature size (Lien and Yuan, 2019). The electric field generated by MEMS-fabricated device electrodes was weak, requiring higher voltages to generate sufficient dielectrophoretic force to capture particles. However, high voltages may lead to negative effects such as Joule heat (Castellanos et al., 2003), potentially damaging the separated biomolecules or the chip itself. Furthermore, MEMS-fabricated devices typically feature channel-based structures with the limited effective surface area, i.e., only the corner in the channel has dielectrophoresis effective, resulting in low efficiency (Fernádez-Morales et al., 2008). In this experiment, we employed a semiconductor CMOS fabrication process to create three-dimensional protruding TiN nano-electrode arrays. Compared to our CMOS-fabricated device, MEMS-fabricated device (Fernádez-Morales et al., 2008) has the following disadvantages: 1) only the electrode tips are effective, leaving most of the chip area devoid of any dielectrophoresis, thus the interdigitated structure i.e. channel structures are utilized to increase tip effectiveness. In contrast, the electrodes of our chip are non-channel structures, so that the entire electrode arrays distributed over almost the entire chip could be used as the effective dielectrophoresis regions; 2) both feature size and uniformity have reached its manufacturing limit, restricting the improvement on the electric field strength; and, 3) large exposed metal areas, nearly 84,000% larger than our CMOS-fabricated electrodes, cause more Joule heat, leading to thermal fluctuations that diminished the dielectrophoresis (Supplementary Figure S1). For this reason, we insulated heat-prone metal lines to avoid direct contacting from medium. We chose TiN, a biologically harmless material, as exposed tip in our chip which is both semiconductor process compatible and biocompatible. Additionally, reducing the electrode size to the nanometer scale not only allowes our CMOS-fabricated electrodes to generate electric fields that are 483.64% (Lien and Yuan, 2019) stronger than the MEMS device but also reduces the exposed area of the electrodes. According to the previous literature (Castellanos et al., 2003), the success of dielectrophoresis has relied on whether the dielectrophoretic force has been greater than the electrothermal force and Brownian motion. The influence of Brownian motion on the particle displacement has not been changed with the voltage, while the influence of electrothermal force has been increased as the voltage has been applied on this specific system. However, as shown in Supplementary Figure S1, only very small area of TiN nano-electrodes on our chip with its insulating layer has been exposed, significantly reducing thermal effects, mainly because the resistance has increased and reduced the power dissipating as heat as well. Moreover, according to throughput requirements, we could implement not only the expansion of chip area but also the scalability. Consequently, our chip has the potential to be developed into commercial products.

According to a global estimate, *Escherichia coli* (*E. coli*) is a common clinical pathogen that can cause a variety of infections, including bloodstream infections, gastrointestinal illness, severe diarrhea, and bacteremia in infants and travellers. The current standard methods for clinical microbial diagnosis involve traditional culture-based enrichment followed by matrix-assisted laser desorption/ionization, time-of-flight mass spectrometry (MALDI-TOF), and polymerase chain reaction (PCR). However, these methods are time-consuming, requiring up to 2–3 days to confirm the final diagnosis, and limited by their inability to distinguish between viable and non-viable bacteria, leading to qualitative errors (Daneman et al., 2023; Fernandez et al., 2017). Dielectrophoresis has emerged as a promising alternative tool for rapid and accurate diagnosis of bacterial infections. This label-free, sensitive, and rapid technique has the potential to detect bacteria at very low concentrations. Dielectrophoresis also has the potential to be used to distinguish between live and dead bacteria. Dielectrophoresis chips have been widely used in diagnostics and clinical research, which can be used to separate bacteria based on their size, shape, and polarizability, e.g., capturing specific bacteria by combining them with antibodies (Yang et al., 2006), and separating live and dead Listeria bacteria by exploiting the differences in their dielectric properties (Bashir and Li, 2002).

In the context of sperm application, the detection of sperm motility is extremely important in pig farms and artificial insemination stations. Currently, the commonly used and more accurate method for sperm motility detection involves computer-assisted devices such as the CASA system or iSperm, which monitor collected sperm quality, noting any samples with less than 70% motility for discard. To increase the effective utilization of sperms requires the employment of a system that not only screens for sperm quality as a means of enhancing fertility and utilization rate, but also reduces costs. It is worth noting that male pigs have poorer meat quality and are less commercially valuable. Male pig meat has a characteristically strong odor that can only be reduced by early castration, which adds an extra step to the rearing process. For this reason, some scientists are looking for ways to separate X and Y chromosome sperms in order to control the gender of piglets produced (Wongtawan et al., 2020). The primary technique for separating X and Y sperms, however, requires an efficient sperm capture process. Traditional sperm separation methods include Swim-up, density centrifugation, and glass wool column. Although these methods are simple and fast, in practical application, insufficient sperm cell recovery or incomplete screening limits their applicability (Pinto et al., 2021). In this experiment, we utilized biocompatible three-dimensional protruding TiN nano-electrode arrays to capture live sperms only and observed the effects of varying voltages and frequencies on sperm cell trapping efficiency. According to the Equations 1, 2, it can be understood that the difference in dielectric constants between the particle and the medium is the key factor determining whether the dielectrophoretic force will work. When the dielectric constants of the particle and the solution become similar, the CM factor approaches to zero, rendering the dielectrophoretic force ineffective. Due to the lytic cell membrane of dead sperm, ions can freely move in and out, causing the dielectric constant of dead sperm to become nearly identical to that of the solution, thereby there are nearly no dielectrophoretic force. However, live sperms with intact cell membranes prevent ions from freely passing through the membrane, and induce effective dielectrophoretic force (van Eijkeren, 2011). Leveraging the different phenomenon of live and dead sperms on dielectrophoretic, we can effectively catch live sperms only with effective dielectrophoretic force.

**FIGURE 1:**
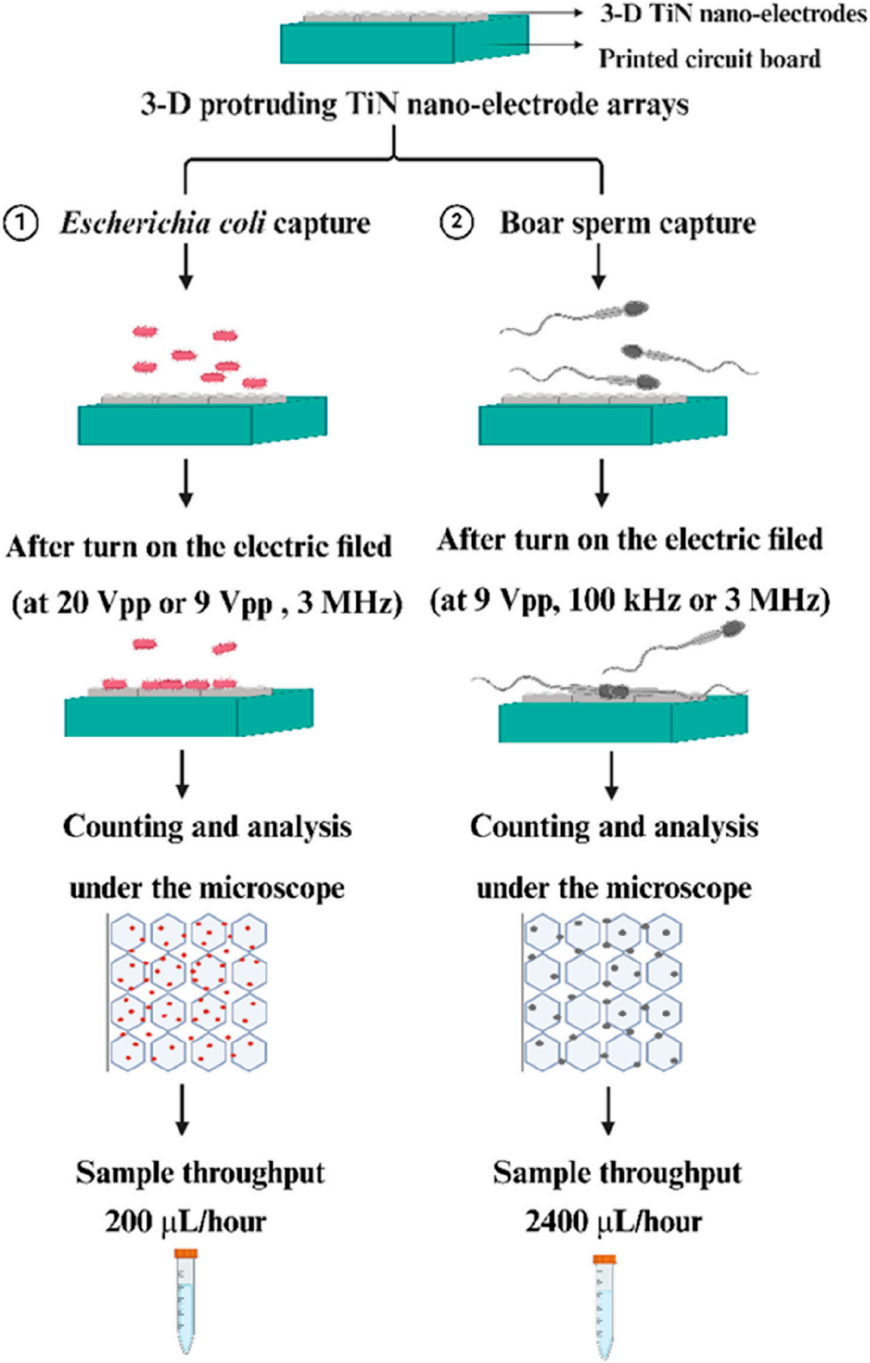
Schematic of our experiments for efficient bacteria and sperm capture through using our 3-D TiN nano-electrode arrays.

In conclusion, the capture efficiency of particles is directly related to the magnitude of the dielectrophoretic force. According to dielectrophoretic theory, as described in Equations 1, 2, this force is varied by several parameters, including the dielectric constant of both the medium and the particle, and the applied AC frequency and voltage (Ronald, 2010; Jones, 1995; Zhang et al., 2019; Jubery et al., 2014). Therefore, understanding how these parameters affect the capture of bacteria and sperm is essential. This study has focused on optimizing these parameters to enhance the capture efficiency of bacteria and sperm using our three-dimensional protruding TiN nano-electrode arrays (as shown in Figure 1).

## Materials and methods

### Fabrication of three-dimensional protruding TiN nano-electrode arrays

**FIGURE 2:**
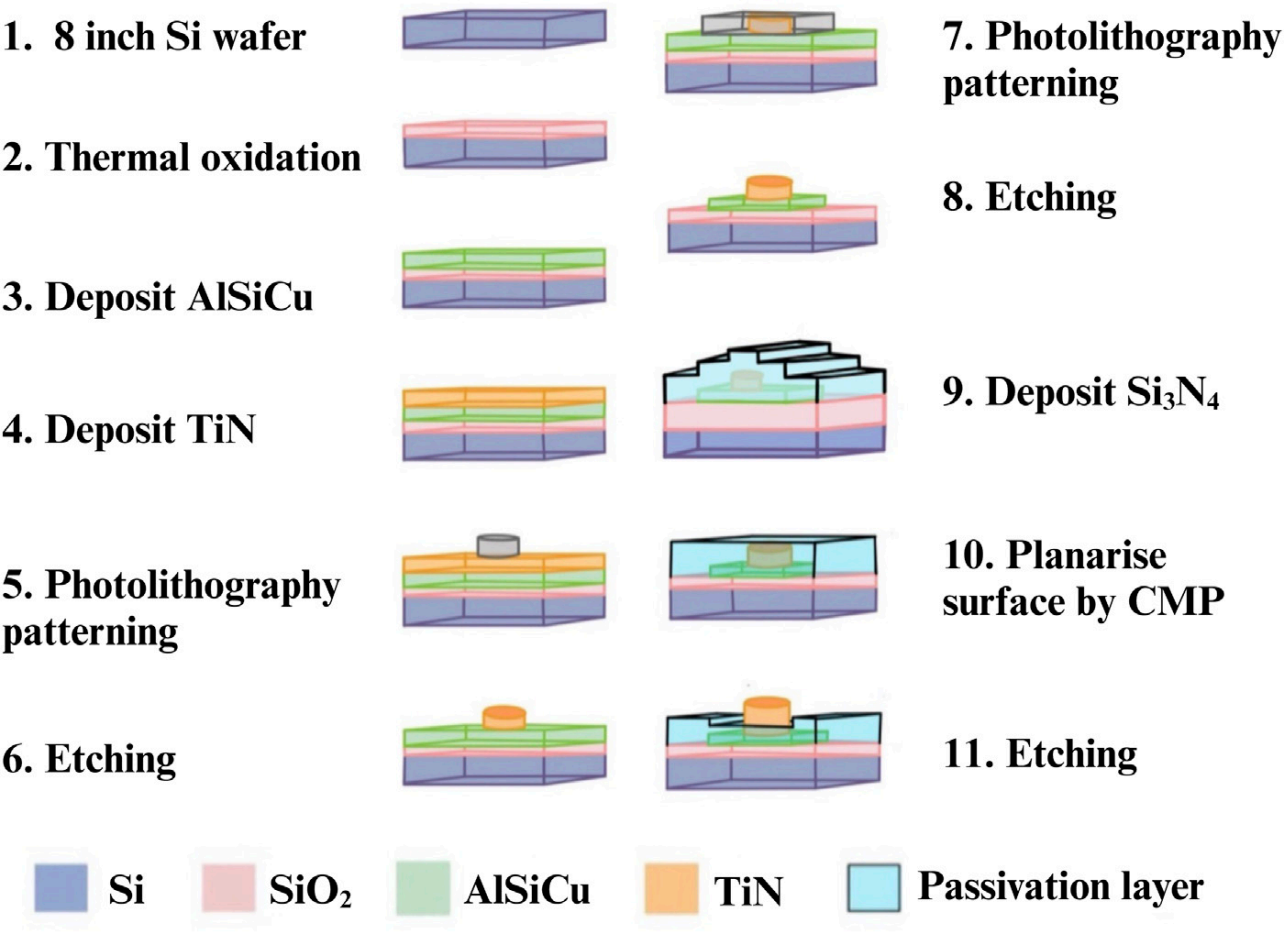
Fabrication of single 3-D protruding TiN nano-electrode.

We followed the chip manufacturing processes of previous precedent (Lien and Yuan, 2019). We used the standard CMOS process with multiple and stacked films, including metal wire (AlSiCu), Titanium nitride (TiN) and passivation (Si3N4), which were precisely aligned and patterned. Figure 2 shows the fabrication process of single electrode. First, a single-crystalline silicon wafer was thermally oxidized to generate a silicon dioxide base layer for isolation. Subsequently, AlSiCu and TiN (electrode material) were deposited on the silicon dioxide via physical vapor deposition (steps 3 and 4). Titanium nitride was chosen as the electrode material due to its chemical stability, corrosion resistance, high conductivity (approximately 8.69 × $10^{6}$ Ω^−1^·m^−1^), good biocompatibility that reduced damage to bacteria and sperms, and lowered the risk of mutual contamination. Photolithography was then used to pattern the electrodes (step 5), followed by dry etching to form the patterned 3-dimensional protruding TiN nano-electrodes (step 6). Steps 7 and 8 patterned the metal lines. After etching and removing the photoresist, a passivation layer of Si_3_N_4_ was applied to the patterned wafer surface (step 9). Finally, the chip surface was planarized via chemical-mechanical planarization (CMP) (step 10), followed by etching of the Si_3_N_4_ layer to: 1) expose the 3-dimensional protruding TiN nano-electrodes; and, 2) bury metal wires under Si_3_N_4_ (step 11). This assured that the patterned TiN electrodes can be exposed to the medium and stood precisely on the metal lines. Furthermore, all metal wires were well shielded. Before all experiments, the chip surface was cleaned with deionized water and 95% alcohol, and dried with a nitrogen gun.

### Calibration and measurement of solution conductivity

We used a Bante540 conductivity meter (Sugar land, United States) to measure solution conductivity, ensuring that the diluted solution was at low conductivity for the experiment. Prior to taking any measurements, the instrument was calibrated using standard solutions with conductivities of 84.0 µS/cm, 1413.0 µS/cm, and 12.88 ms/cm, respectively. We then soaked the detector probe in the measured solution. After each measurement, we washed the probe with deionized water three times.

### Bacteria culture and preparation

*Escherichia coli* (*E. coli*) is a gram-negative, rod-shaped bacterium commonly found in the human gut microbiota. The specific DH5α strain of *E. coli* used in this study was procured from the Bioresource Collection and Research Center (Hsinchu, Taiwan). The *E. coli* strain was initially inoculated into a 5 mL volume of tryptic soy broth (TSB) medium (T8907-500G, Sigma-Aldrich, United States) and incubated at a controlled temperature of 37°C for 24 h. Following this initial growth phase, a 10 µL aliquot of the bacteria culture was retrieved and plated onto a solidified TSB agar plate. The *E. coli* culture on the agar plate was then incubated for an additional 24 h at 37°C. Subsequently, well-isolated bacteria colonies were harvested from the TSB agar plate and resuspended in deionized water. This process resulted in a bacteria suspension with an estimated concentration of 2 × 10^8^ colony forming units per millilitre (CFU/mL), which was verified by measuring the optical density of the suspension at a wavelength of 600 nm. In addition, because the conductivity of the medium also affects the magnitude of the dielectrophoretic force, we also tested the use of medium with different conductivities to culture *E. coli.* We investigated whether each medium was conducive to bacteria growth or harmful to bacteria (Supplementary Figure S2).

### Sperm preparation

Using Landrace breed boar semen purchased from the Animal Technology Institute at the Agricultural Technology Research Institute (Miaoli, Taiwan). Semen was placed in a 37°C water bath for 20 min to equilibrate, then the iSperm system (Aidmics Biotechnology, Taipei, Taiwan) was used to test sperm cell concentration and sperm cell motility. Semen was diluted slowly with dielectrophoresis buffer to a concentration of 7 × 10^6^ sperm/mL. The dielectrophoresis buffer contained 10 mM HEPES, 0.1 mM CaCl_2_, 236 mM sucrose, and 59 mM dextrose, and had a pH level of 7.4 (Pendharkar et al., 2022).

### Statistical analysis

This experiment employed GraphPad Prism version 8.4 (GraphPad Software, CA, United States) to conduct statistical analysis on the data. The capture efficiency of *E. coli* was analyzed using Student’s t-test. Experimental data from various concentrations are depicted as bar charts, with the horizontal axis representing *E. coli* concentration (CFU/mL) and the vertical axis indicating bacteria capture efficiency (%). For the analysis of sperm capture efficiency, one-way ANOVA was employed to determine significant differences between groups. Experimental data from different conditions are presented as bar charts, with the horizontal axis representing the applied peak-to-peak voltage (Vpp) and the vertical axis indicating sperm capture efficiency (%). A *p*-value less than 0.05 was considered statistically significant in both above experimental analysis methods.

## Results and discussion

### Structure of three-dimensional protruding TiN nano-electrode arrays

**FIGURE 3:**
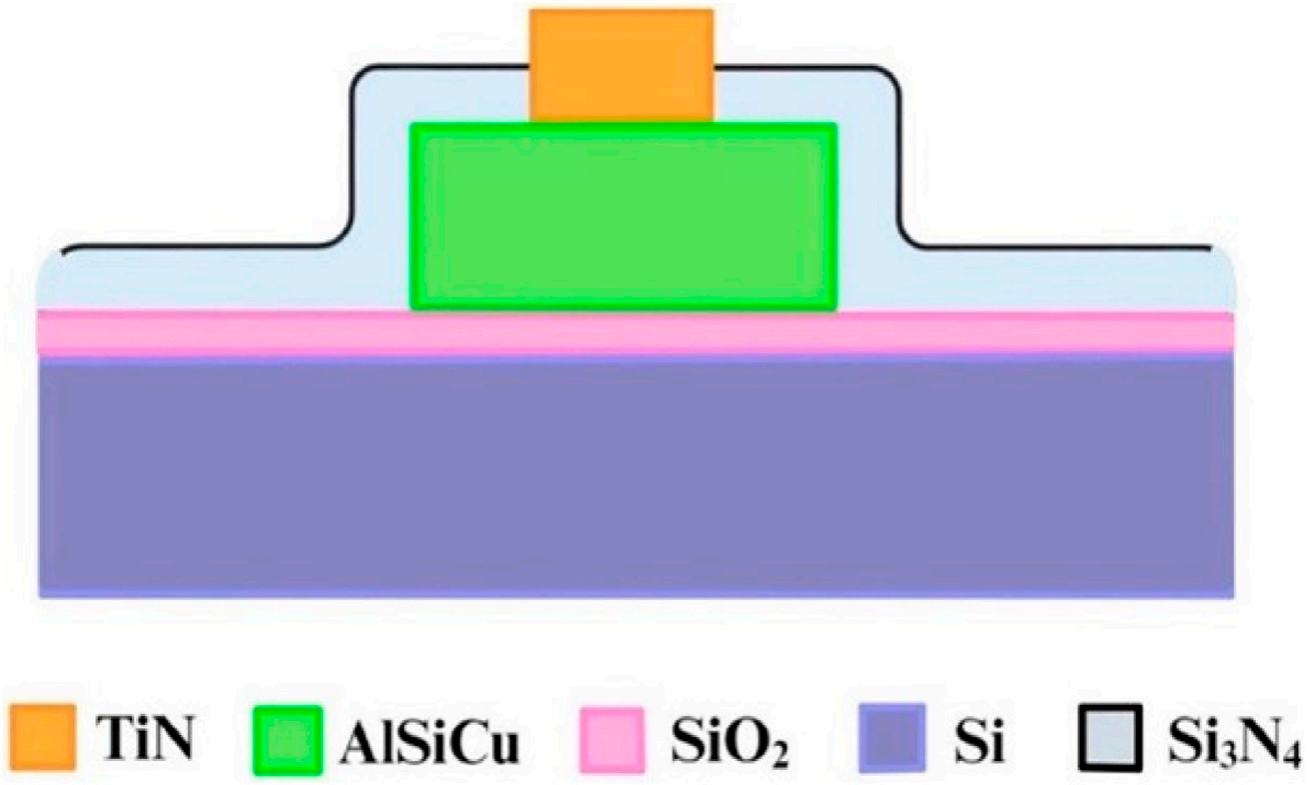
Cross-sectional schematic of single three-dimensional protruding TiN nano-electrode.

Figure 3 provides a cross-sectional diagram. Because the wires (AlSiCu) in this array are non-biocompatible, a passivation layer (Si_3_N_4_) was used to bury them. This not only reduced direct harm to bacteria or sperm but also isolated the Joule heat generated by the wires, thereby minimizing thermal fluctuation interference with dielectrophoretic force. This allowed the dielectrophoretic force to function more effectively.

**FIGURE 4:**
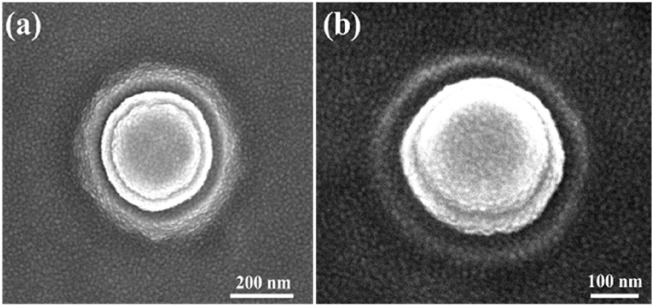
Images of single nano-electrode captured by scanning electron microscopy: **(A)** top view of a nano-electrode and **(B)** 20° tilt view of a nano-electrode.

The nano-electrode structures were designed with 3-dimensional protruding cylindrical shapes, with a diameter of approximately 200 nm, as shown in Figure 4, which significantly increased the electric field strength. The top and tilt view images captured by scanning electron microscopy are shown in Figures 4A, B, respectively.

**FIGURE 5:**
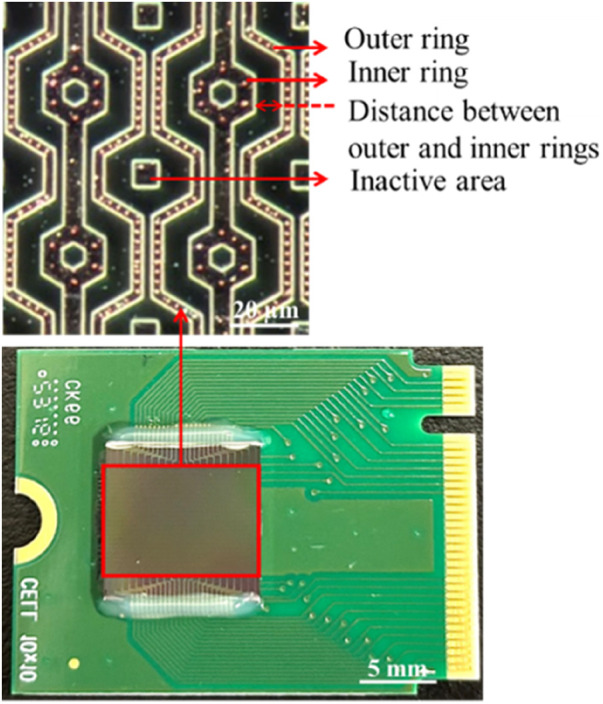
3-D protruding TiN nano-electrode chip. The electrode array observed under a microscope with the dark field.

**FIGURE 6:**
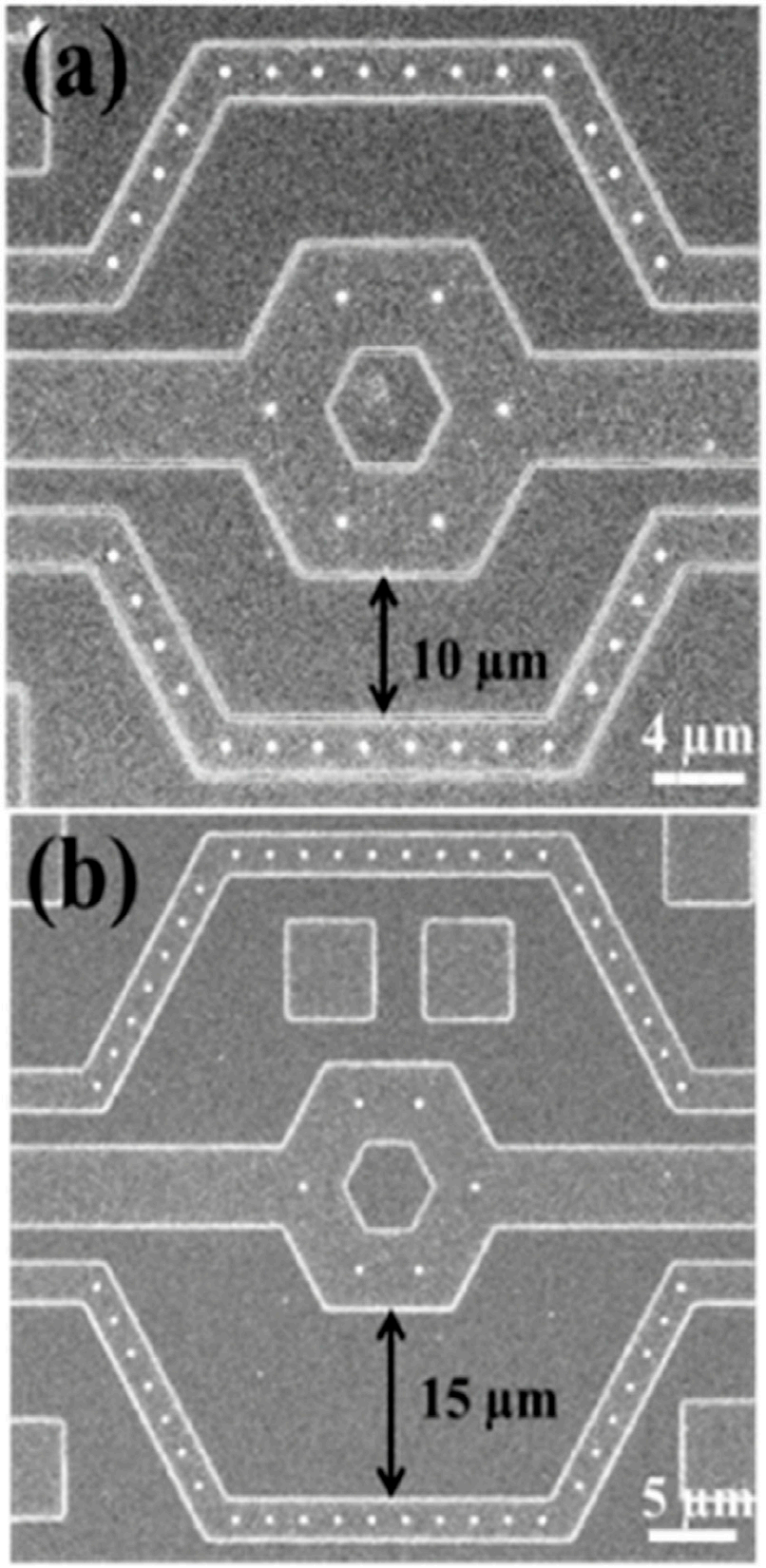
Scanning electron microscopy images of nano-electrode arrays. Three-dimensional protruding nano-electrode arrays have distances of **(A)** 10 µm and **(B)** 15 µm between the inner and outer rings, respectively.

**FIGURE 7:**
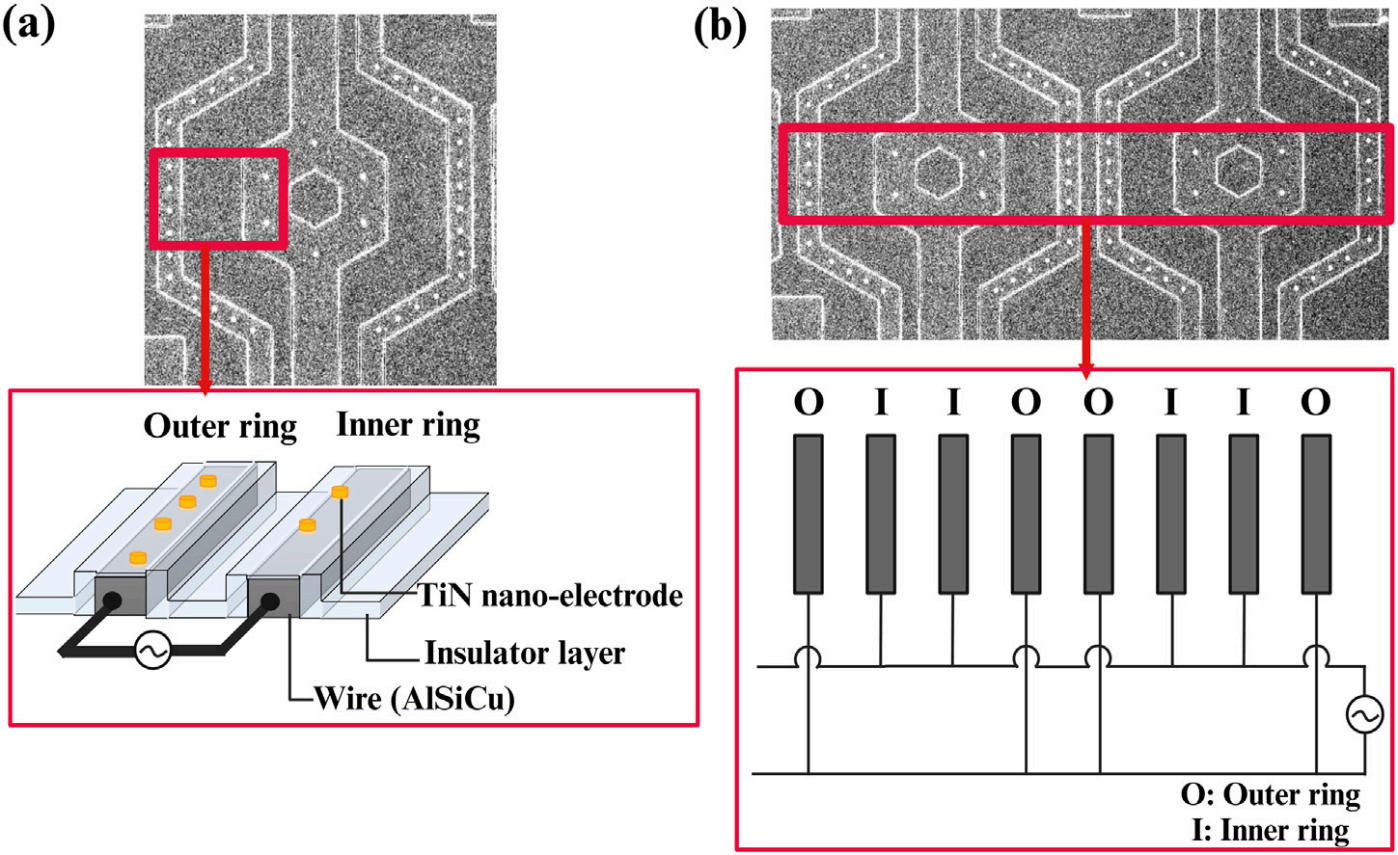
Schematic of the electrical connections for 3D protruding TiN nano-electrode arrays; **(A)** a unit block electrical connection with wires insulated and only TiN nano-electrodes exposed and **(B)** electrical connections between blocks.

Moreover, the electrode array was arranged in a unit hexagon block to ensure that the distances between electrodes were nearly equal, as shown in Figure 5. The region outside the array was designated as an inactive area, as shown in Figure 5. Furthermore, the selection of distance between the inner and outer rings of the 3-dimensional protruding nano-electrode arrays adaptively matched the size of the target object to avoid the possibility of adjacent unit block spillover resulting from an overly small separation distance. For our bacteria and sperm experiments, we utilized a distance of 10 and 15 µm respectively (Figures 6A, B). All electrical connections are shown in Figure 7. All inner ring electrodes were connected to each other, and all outer ring electrodes were connected to each other, and an alternating current was provided between the inner and outer rings.

### Conductivity measurement

Solution conductivity affects the magnitude of the dielectrophoretic force. If the solution conductivity is high, the smaller dielectrophoretic force is obtained. Therefore, it was necessary to dilute the solution to a level suitable for separating the target biological samples using low conductivity. Following calibration, the measurement probe was immersed in various solutions, including deionized water, bacteria suspensions, and sperm solutions, to determine their conductivities (Table 1).

**TABLE 1:** Measured conductivity of solutions.

Sample	Conductivity
Deionized water	0.55−0.70 µS/cm
*E. coli* suspension in deionized water (1 × 10^8^ CFU/mL)	100 µS/cm
Stocked sperm solution	7.00−8.00 mS/cm
Diluted sperm solution in dielectrophoresis buffer (7 × 10^6^ sperm/mL)	700−800 µS/cm
Dieletrophoresis buffer	307 µS/cm

### Dielectrophoretic capture of live *Escherichia coli* in deionized water

**FIGURE 8:**
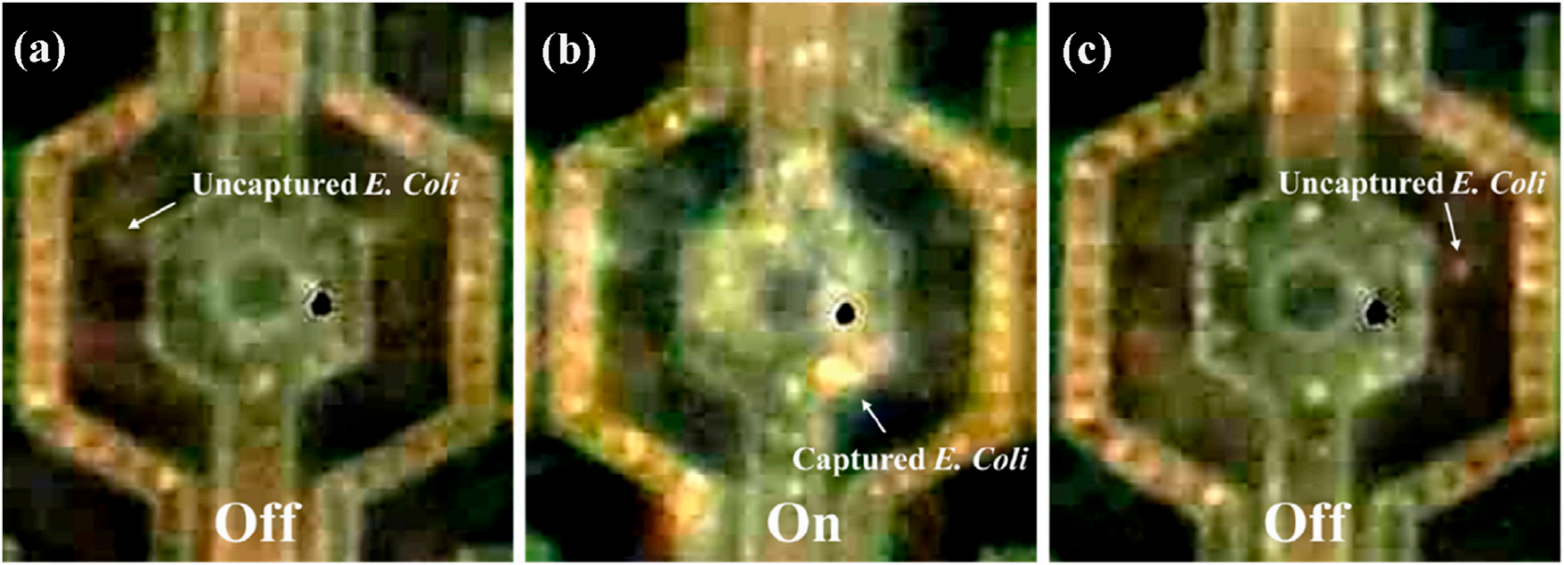
Behavior of live *Escherichia coli* being attracted to the nano-electrodes by dielectrophoretic force. **(A)** *E. coli* randomly separated on the chip at the original state (no electric field). **(B)** *E. coli* bacteria were attracted to the nano-electrodes by dielectrophoretic force at 9 Vpp, 3 MHz. **(C)** *E. coli* bacteria were no longer attracted to the chip when the electric field was turned off.

This experiment investigated the ability of our chip to capture and release live *E. coli.* A bacteria suspension with a concentration of 1.8 × 10^8^ CFU/mL was prepared. We first dropped a 10 µL bacteria suspension onto our chip, covered it with a coverslip and then generated a sine wave signal using a function generator (MFG-2260MFA, Gwinstek, New Taipei City, Taiwan) as the alternating current signal source to provide a continuous sinusoidal signal. This signal had a frequency of 3 MHz and a peak-to-peak voltage of 9 Vpp. Figure 8 illustrates that, under baseline conditions (no electric field applied), the number of *E. coli* adhering to the electrodes was relatively low. However, when a 9 Vpp, 3 MHz electric field was activated for 3 min, a significant increase in bacteria adhesion to the electrodes was observed. This suggests a positive dielectrophoresis effect, where the electric field induces a polarization in the *E. coli*, attracting them towards the high-field regions at the nanoelectrodes. Following the three-min electric field exposure, the field was deactivated for another 3 min. After deactivation, a noticeable increase in the free-floating bacteria population was observed, suggesting the release of captured *E. coli* from the electrodes (Supplementary Movies S1, S2). This cyclical capture and release behavior demonstrates the potential utility of the 3-dimensional protruding TiN nano-electrode arrays for manipulating and analyzing live *E. coli*.

### Quantifying the efficiency of dielectrophoretic capture of live *Escherichia coli*

**FIGURE 9:**
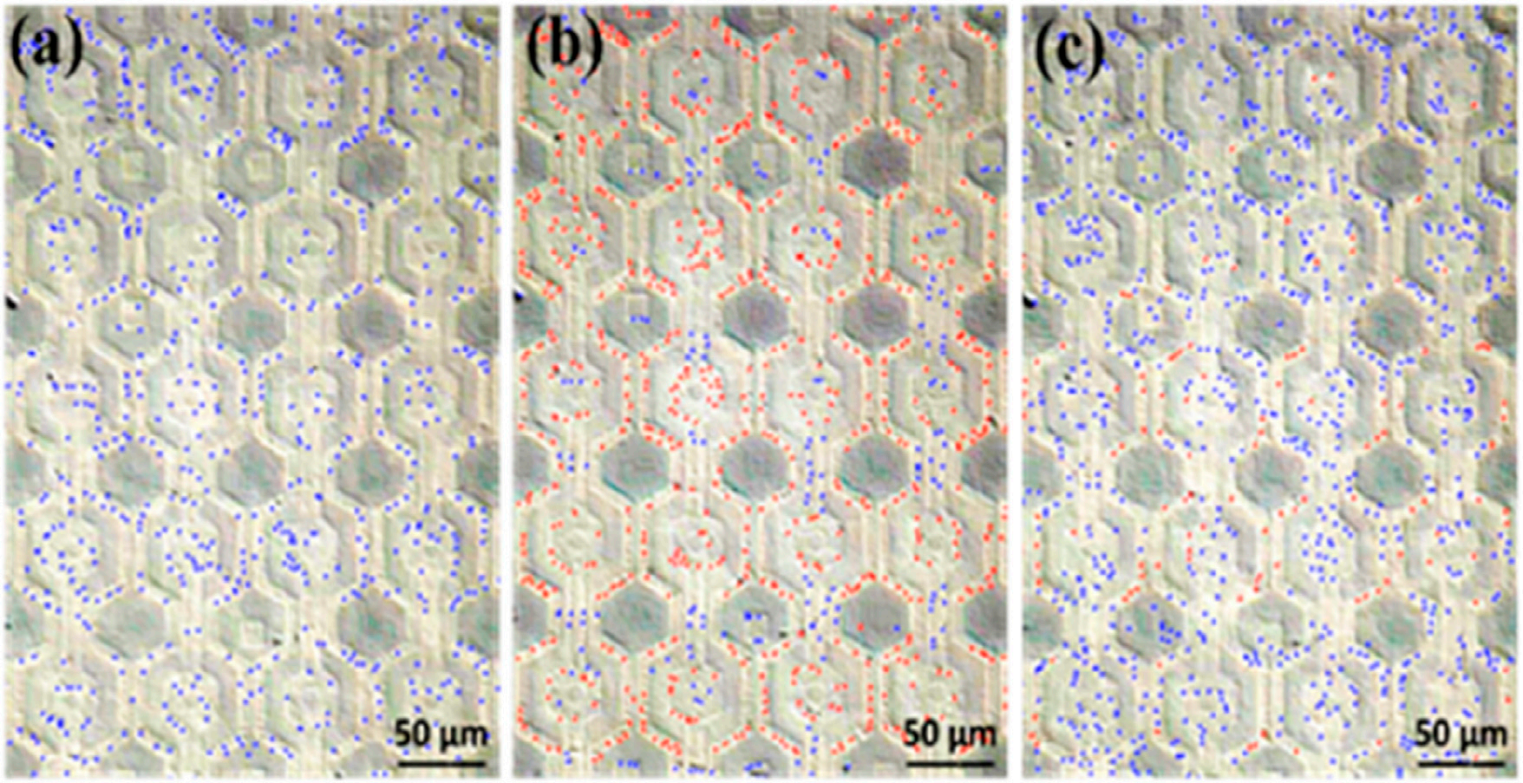
Count of free-floating and captured *E. coli* (with free-floating bacteria marked in blue and captured bacteria in red). **(A)** At the original state, with the electric field off, *E. coli* bacteria were observed to be randomly distributed. **(B)** Upon activating the electric field at 20 Vpp, 3 MHz, the captured *E. coli* predominantly concentrate around our nano-electrodes. **(C)** After deactivating the electric field, most of the bacteria return to a random distribution, although a minority remain fixed near the nano-electrodes. This is speculated to be due to either clusters of bacteria that were less able to escape or individual bacteria with diminished vitality making them more likely to remain adhered.

*E. coli* suspensions were prepared with estimated concentrations of 2.5 × 10^8^ CFU/mL and 1.75 × 10^8^ CFU/mL. The conductivity of the deionized water ranged between 0.55 and 0.70 μS/cm, while that of the bacteria suspension was approximately 100 μS/cm. After applying the *E. coli* suspension onto the dielectrophoresis chip and then applying an electric field with a voltage of 20 Vpp at a frequency of 3 MHz, the bacteria experienced positive dielectrophoretic force, leading to aggregate at electrodes where have higher electric field, as shown in Figure 9. The calculation of *E. coli* in Figure 9 was performed during microscopic observation. In this figure, the red and blue dots were manually marked using the MicrocamV8 software after video observation under the microscope. The determination was based on the dynamic behavior of the bacteria. *E. coli* attracted to the nano-electrodes and ceased movement were marked as red dots, while those not captured were marked as blue dots. In Figure 9A, without an electric field, all *E. coli* were classified as floating (not affected by dielectrophoresis) and marked as blue dots. In Figure 9B, after the electric field was applied, some *E. coli* was attracted to the nano-electrodes and stopped moving, marked as red dots (captured *E. coli*), while the remaining floating bacteria were still marked as blue dots. In Figure 9C, after the electric field was turned off, most bacteria resumed random movement and were again marked as blue dots. Due to clustering, which made it difficult for them to move, a few bacteria that remained attached to the nano-electrode surfaces were marked as red dots. Furthemore, to verify whether capture efficiency varied across different bacteria concentrations, the procedure for these two bacteria concentrations was replicated four times under conditions of a 3 MHz frequency and 20 Vpp. Before each repetition, the chip was sequentially rinsed with deionized water 3–5 times and then with 95% alcohol to ensure that there was no cross-contamination between experiments. The results of each count for these two bacteria concentrations are detailed in Supplementary Tables S1, S2, respectively. In calculating the capture efficiency after electric field activation, we observed that captured bacteria sometimes formed clusters, particularly around the nano-electrodes. These clusters, potentially containing several bacteria, could be counted as a single unit, leading to an underestimation of the true capture efficiency based on individual bacteria. Consequently, we hypothesize that the difference between the total number of bacteria in the original state and the number of free-floating bacteria after the electric field application may provide a closer estimate to the actual number of bacteria captured as defined following equation.

$$Dielectrophoretic\;capture\;efficiency\;of\;E.\;coli\;\left(\%\right)=\frac{\begin{array}{c} \left(Number\;of\;E.coli\;at\;the\;original\;state\right)-\\ \left(Number\;of\;floating\;E.\;coli\;after\;turning\;on\;the\;electric\;field\right) \end{array}}{\left(Number\;of\;E.\;coli\;at\;the\;original\;state\right)}\times 100\%$$

**FIGURE 10:**
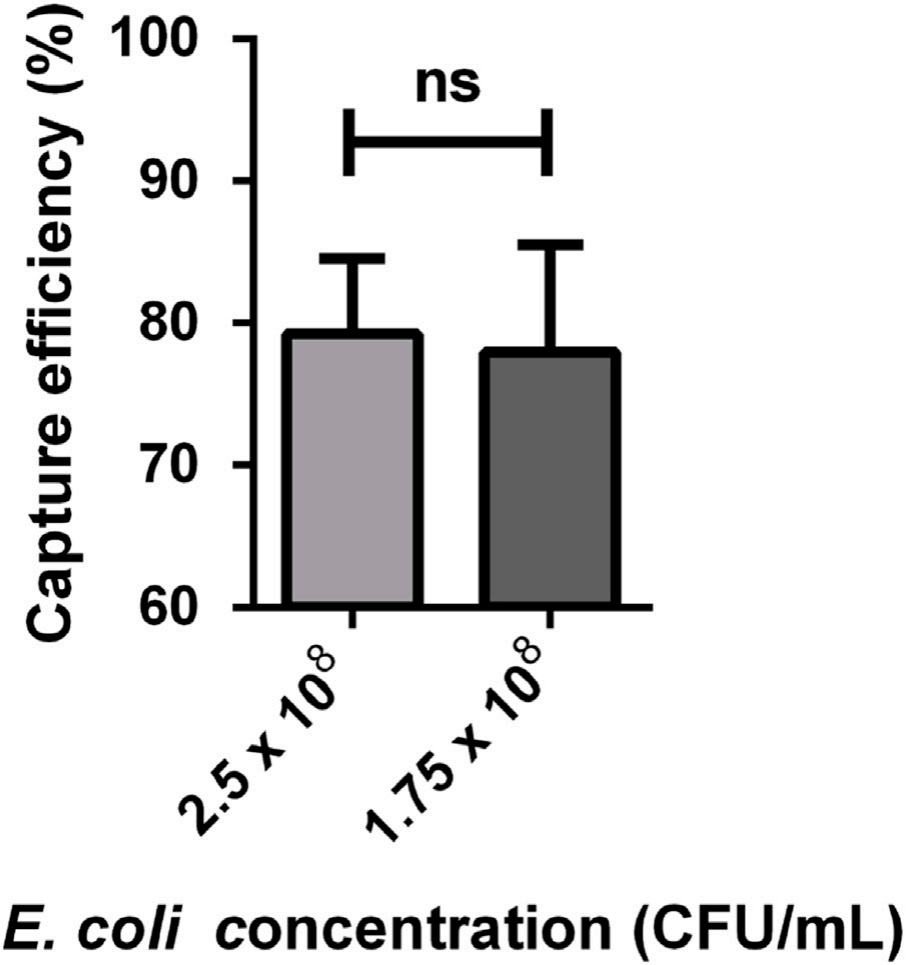
*E. coli* capture efficiency at different concentrations (n = 4). The data was analysed using Student’s t-test. The capture efficiency at a bacteria concentration of 2.5 × 10^8^ and 1.75 × 10^8^ CFU/mL was 79.25% ± 2.66% and 77.95% ± 3.78%. ns, no significant.

Based on the capture efficiency data for each bacteria concentration (Supplementary Tables S1, S2), it was calculated that the average capture efficiencies were approximately 79.25% ± 2.66% and 77.95% ± 3.78% respectively. Next, we adopted the Student’s t-test to analyze capture efficiency at different concentrations, and the results indicated no significant difference. This suggests that the capture efficiency did not vary significantly between these two bacteria concentrations, as shown in Figure 10.

In conventional MEMS electrodes, only corners or tips are effective dielectrophoresis regions (Lien and Yuan, 2019; Fernádez-Morales et al., 2008). The channel structure is designed to increase the quantity of effective dielectrophoresis regions and the liquid primarily flows through channels to ensure the liquid passes through effective dielectrophoresis regions (i.e., the electrode corner or tip). However, the electrodes of our chip are non-channel structures, so that the entire electrode arrays distributed over almost the entire chip could be used as the effective dielectrophoresis regions. Regarding the throughput, our estimated value: in a 3-min dielectrophoresis capture test, we have achieved a satisfactory capture efficiency of bacteria mixed in deionized water approximately 79.25% ± 2.66% through processing 10 μL of the sample with a 3-min dielectrophoresis on operation. Based on this, we have estimated that 200 μL of the sample can be handled within 1 h. Moreover, due to the scalability of our chip, higher throughput can be achieved based on requirements. Around 3–10 mL specimen is required in clincal pratice for bacterial diagnosis. Consequently, scalability demonstrates the considerable potential for further advancements in our 3D protruding TiN nano-electrode arrays for the applications of bacterial diagnosis.

### Dielectrophoretic capture of live sperms in three-dimensional protruding TiN nano-electrode arrays

We first dropped 20 µL of diluted semen onto our chip, covered it with a coverslip and then generated a sine wave signal using a function generator (MFG-2260MFA, Gwinstek, New Taipei City, Taiwan). We used a recordable microscope (OLYMPUS MX50, Japan) to observe the movement of sperm under different frequencies and voltages. We utilized the MicroCamV8 software provided by M&T OPTICS (Taipei, Taiwan) to calculate the number of sperms exhibiting dielectrophoretic phenomena, and then computed the dielectrophoretic capture efficiency using the following equation. The captured sperm were highlighted through video editing and then counted using MicrocamV8. We counted all sperm exhibiting dielectrophoretic phenomenon during the period when the electric field was activated. The capture efficiency was calculated by dividing the number of sperm captured within the effective electric field by the total number of sperm observed in the video.

$$Dielectrophoretic\;capture\;efficiency\;of\;sperm\;\left(\%\right)=\frac{\left(Number\;of\;captured\;sperms\;in\;dielectrophoretic\;buffer\right)}{\left(Total\;live\;sperms\;in\;dielectrophoretic\;buffer\right)}\times 100\%$$

When the function generator was activated, sperms that moved closer to the electrodes were considered to be sperms affected by dielectrophoretic force (Supplementary Movie S3).

### Quantifying the efficiency of dielectrophoretic capture of live sperms

**FIGURE 11:**
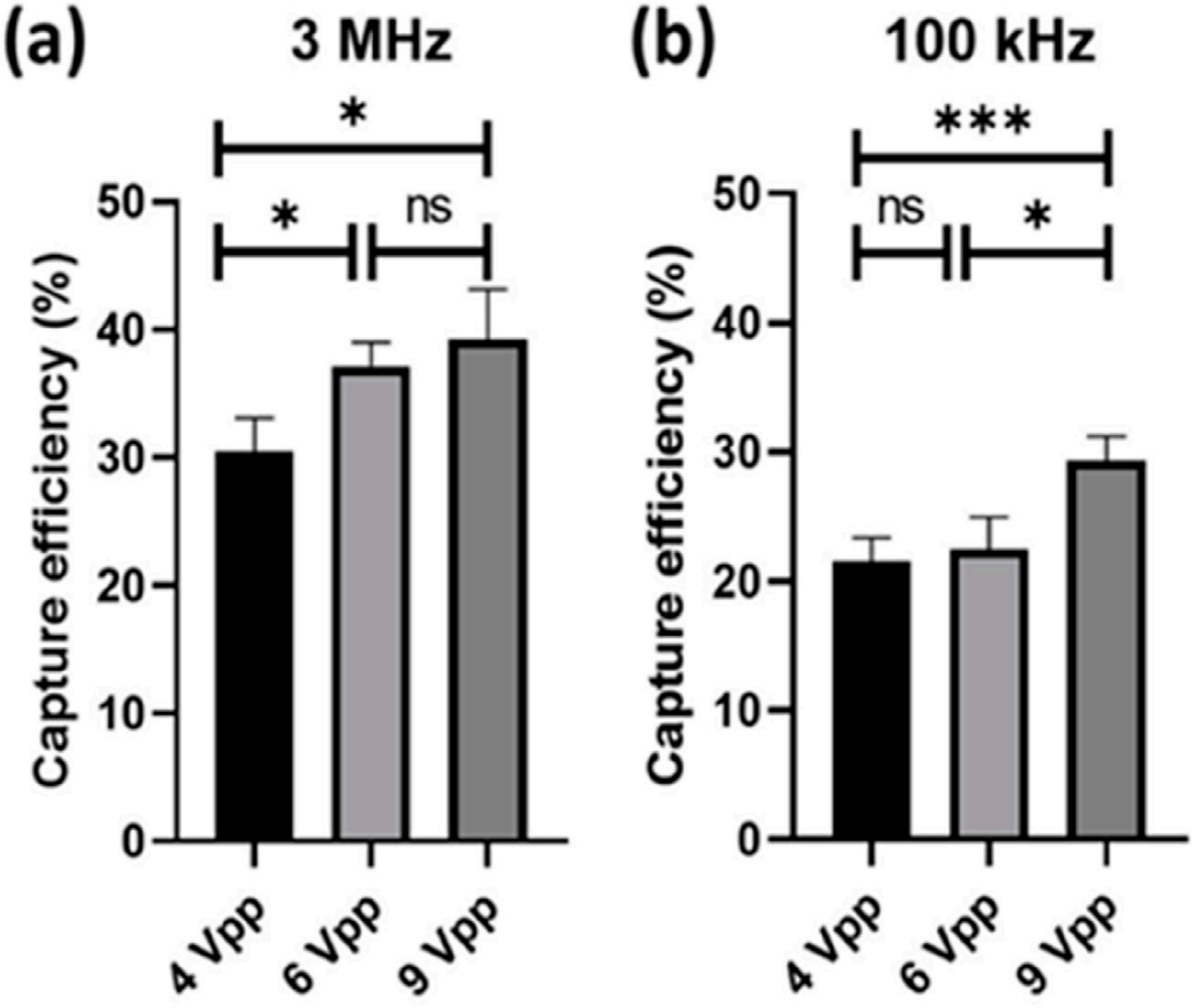
Sperm capture efficiency at different frequencies and voltages (n = 5). The data was analysed using one-way ANOVA. **(A)** Sperm capture efficiency at 4 Vpp (30.5 ± 2.6), 6 Vpp (37.1 ± 1.9), and 9 Vpp (39.2 ± 3.9) under 3 MHz. **(B)** Sperm capture efficiency at 4 Vpp (21.6 ± 1.8), 6 Vpp (22.5 ± 2.5), and 9 Vpp (29.3 ± 1.9) under 100 kHz. *, 0.01 < *P* < 0.05; ***, *P* < 0.001; ns, no significant.

We conducted tests on the sperm capture efficiency at different voltages under frequencies of 3 MHz and 100 kHz. As depicted in Figure 11A, at a frequency of 3 MHz, the sperms capture efficiency for 4, 6, and 9 Vpp mostly ranged between 30% and 40%. There are significant differences between 4 and 6 Vpp, as well as between 4 and 9 Vpp. In Figure 11B, it can be observed that the sperm capture efficiency under this condition was approximately between 20% and 30%, with significant differences in sperm cell capture efficiency between 4 and 9 Vpp, as well as between 6 and 9 Vpp. Additionally, it can be observed that the capture efficiency was generally higher at 3 MHz compared to 100 kHz. According to the dielectrophoretic force equaiton aforementioned, the frequency is a varient of the Clausius-Mossotti factor, thereby influences the dielectrophoretic force. Our experimental results show that the capture efficiency of boar sperm with conductivity 0.08 S/m is better at 3 MHz, which is comparable to the simulation (Park et al., 2020).

Many studies have shown that dielectrophoresis is effective for sperm selection (Bashir and Li, 2002; Yang et al., 2006; Wongtawan et al., 2020; Rosales-Cruzaley et al., 2013), and can even increase the concentration of sperm near the ovum to improve the chances of fertilization (Kao, 2014). However, due to limitations in chip fabrication, throughput has always been a significant challenge, thereby limiting its efficiency and feasibility in practical applications. From our results by using 3-dimensional-TiN nano-electrode arrays for sperm capture, it is feasible to process 2,400 μL of semen per hour, with a maximum capture efficiency of 39.2% ± 3.9%. Compared to previous devices, our approach is faster and can handle a larger volume of samples (Dararatana et al., 2015). Additionally, depending on the amount of semen to be processed, we could enlarge the area or increase the quantity of the chip for parallel processing. However, due to the potentially differentiation of dielectrophoretic force on the head and tail of sperms (Shuchat et al., 2019), the current chip with an inner and outer electrode distance of 15 µm may not match the size of sperms. This leads to the head of sperms being captured by the nano-electrodes while the tail fluttered in multiple electric field regions, and potentially reduces the efficiency of sperms capture in non-uniform electric fields.

## Conclusion

In this study, we adopted a semiconductor CMOS fabrication process. This approach not only isolates heat-prone metal lines and biologically harmful materials, but also utilizes nano-scale electrodes to augment the strength of the electric field compared to MEMS fabrication. This approach not only isolates heat-prone metal lines and biologically harmful materials but also utilizes nanoscale electrodes to augment the strength of the electric field. As a result, Particles can be captured at lower voltages, and is also feasible at a high voltage of 20 Vpp. Furthermore, to meet throughput requirements, the chip processing area can be expanded and scalability can be implemented, presenting potential opportunities for the development of commercial products. With our current chip, we can process approximately 200 μL of bacteria samples per hour and 2,400 μL of sperm samples per hour.

To capture bacteria using dielectrophoretic force, we applied a voltage of 20 Vpp and a frequency of 3 MHz, observing significant dielectrophoresis behavior that facilitated efficient bacteria capture. Quantitative analysis revealed a maxium capture efficiency of approximately 79.25% ± 2.66% following a three-min electric field activation. In terms of sperm capture, the efficiency at 3 MHz was higher than at 100 kHz and higher applied voltage get higher efficiency. Sperm capture efficiency were approximately between 20% and 40%.

Overall, 3-dimensional protruding TiN nano-electrode arrays offers notable advantages such as the potential of mass production, cost-effectiveness, and applications in bacteria culturing and sperms selecting. In the future, efforts will be focused on adjusting various parameters for bacteria and sperm capture, such as conductivity, frequency, and voltage, to enhance capture efficiency.

## Data Availability

The original contributions presented in the study are included in the article/Supplementary Material, further inquiries can be directed to the corresponding authors.
