# Supplementary material for: Dielectrophoretic capture of Escherichia coli and boar sperms using ULSI-fabricated three-dimensional protruding TiN nano-electrode arrays
Source: Front Bioeng Biotechnol. 2024 Oct 1;12:1470606. doi: 10.3389/fbioe.2024.1470606 (PMC11473421; doi:10.3389/fbioe.2024.1470606)
Supplement: Supplementary file 4 [file DataSheet1.docx]

Supplementary Material

Dielectrophoretic capture of *Escherichia coli* and boar sperms using ULSI-fabricated three-dimensional protruding TiN nano-electrode arrays

Hua-Jung Lu^a#^, I-Hsuan Liao^a#^, Chun-Lung Lien^b^, Jeng-Huei Shiau^b^, Ching-Fen Shen^c^, Kuan-Ru Chou^b*^ Chao-Min Cheng^a*^

^a^Institute of Biomedical Engineering, National Tsing Hua University, Hsinchu, Taiwan

^b^NEAT Biotech Inc., Hsinchu, Taiwan

^c^Department of Pediatrics, National Cheng Kung University Hospital, College of Medicine, National Cheng Kung University, Tainan, Taiwan

^#^These authors equally contributed.

^*^Corresponding author: [chaomin@mx.nthu.edu.tw](mailto:chaomin@mx.nthu.edu.tw) (to C.-M.C.) & [krchou@neat-bio.com](mailto:krchou@neat-bio.com) (to K.-R.C.)

#
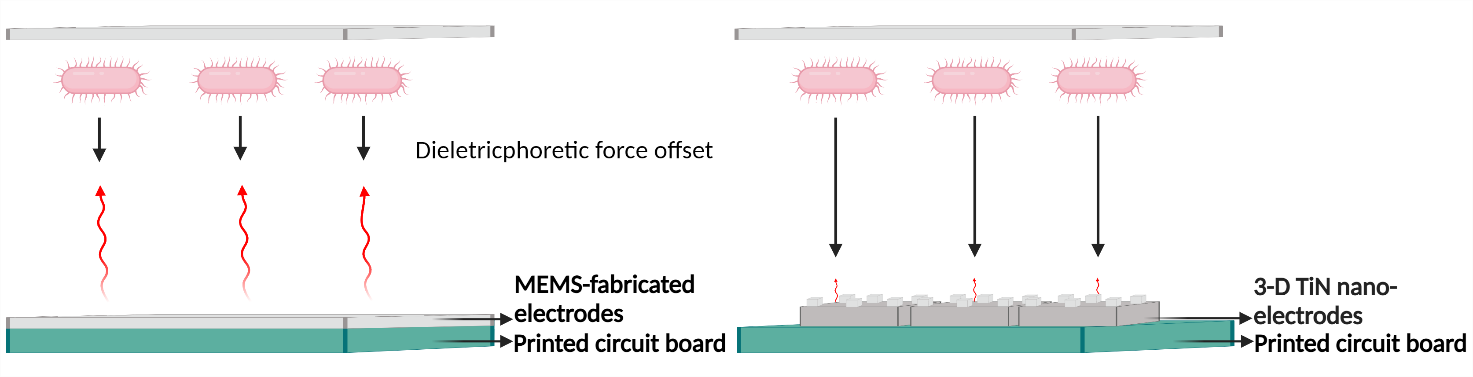


# Figure S1 Schematic of thermal fluctuations induced by (a) MEMS-fabricated electrodes and (b) our CMOS-fabricated TiN nano-electrodes. When dielectrophoresis is active, the large exposed metal lines of MEMS-fabricated electrodes directly contact the medium, causing Joule heat. The Joule heat would induce thermal fluctuations due to the temperature gradient, creating an upward force that opposes and weakens the dielectrophoretic force. By contrast, our nano-electrode-based chip has incorporated insulated metal lines, reducing both direct contact with the medium and Joule heat. This would minimize thermal fluctuations that would offset the dielectrophoretic force, and increase the net pulling force as well.

# Impact of background solutions on *E. coli* growth

# To optimize chip capture efficiency downstream, it is critical to evaluate not only the conductivity of the background solution but also its influence on *E. coli* growth. Four solutions were investigated: tryptic soy broth (TSB), dielectrophoresis (DEP) buffer, a 1:1 mixture of TSB and DEP buffer (mix), and phosphate-buffered saline (PBS). An *E. coli* suspension with an initial concentration of 1.8×10^8^ CFU/mL was prepared and verified by measuring OD600. Subsequently, 0.5 mL aliquots of the *E. coli* suspension were inoculated separately into 4.5 mL of each solution (TSB, DEP, mix, PBS) in individual culture tubes. These cultures served as the experimental groups and were incubated at 37°C for 24 and 48 hours. To assess viability throughout the experiment, two control groups were established for each solution. Each control group contained 5 mL of the corresponding solution (TSB, DEP, mix, PBS) in a separate culture tube. One control group for each solution was incubated at 37°C, while the other was incubated at room temperature 25°C. After 24 and 48 hours of incubation, the bacterial concentration in each group was measured using OD600. Figure S1 demonstrates that, regardless of the incubation time (24 or 48 hours), the measured *E. coli* concentrations followed this order: TSB > Mix > PBS > DEP. TSB supported the highest *E. coli* growth, exceeding even the initial bacterial concentration, indicating optimal growth conditions. Conversely, cultures in other solutions exhibited a decrease in concentration compared to the initial inoculum. Therefore, TSB was chosen as the preferred background solution for subsequent experiments.


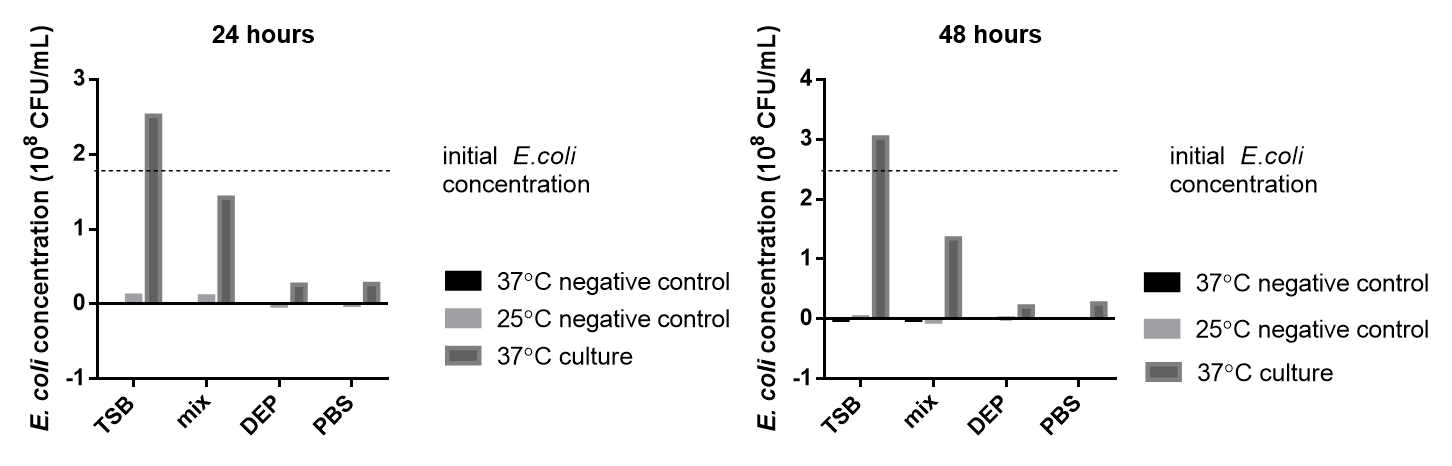


**Figure S2** Variation in *E. coli* concentration under different solution incubations. Regardless of the 24 hours or 48 hours culture period, the measured *E. coli* concentrations were ranked as follows: TSB > Mix > PBS > DEP. In TSB medium, the concentration was the highest, indicating optimal growth conditions for *E. coli*, even surpassing the initial bacterial liquid concentration. By contrast, cultures in other solutions showed a decrease in concentration compared to the initial one.

**Table S1** Repeated experiments on counting and capture efficiency of *E. coli* captured by dielectrophoresis at a concentration of 2.5×10^8^ CFU/mL.

| Sample | Before DEP turn on  (bacteria number) | DEP turn on  (floating bacteria number) | DEP turn on  (captured bacteria number) | Capture efficiency (%) |
| --- | --- | --- | --- | --- |
| *E.coli*-1 | 2633 | 334 | 895 | >87% |
| *E.coli*-2 | 1779 | 394 | 1829 | >78% |
| *E.coli*-3 | 1707 | 382 | 1610 | >77% |
| *E.coli*-4 | 2018 | 502 | 1615 | >75% |

**Table S2** Repeated experiments on counting and capture efficiency of *E. coli* captured by dielectrophoresis at a concentration of 1.75×10^8^ CFU/mL.

| Sample | Before DEP turn on  (bacteria number) | DEP turn on  (floating bacteria number) | DEP turn on  (captured bacteria number) | Capture efficiency (%) |
| --- | --- | --- | --- | --- |
| *E.coli*-1 | 1018 | 191 | 927 | >81% |
| *E.coli*-2 | 927 | 197 | 1001 | >86% |
| *E.coli*-3 | 1219 | 297 | 983 | >76% |
| *E.coli*-4 | 1235 | 386 | 874 | >69% |
